# Dartagnan: Bounded Model Checking for Weak Memory Models (Competition Contribution)

**DOI:** 10.1007/978-3-030-45237-7_24

**Published:** 2020-03-13

**Authors:** Hernán Ponce-de-León, Florian Furbach, Keijo Heljanko, Roland Meyer

**Affiliations:** 8 Johannes Kepler University, Linz, Austria; 9 University of Birmingham, Birmingham, UK; 10 University of the Bundeswehr Munich, Munich, Germany; 11 TU Braunschweig, Braunschweig, Germany; 12 University of Helsinki and HIIT, Helsinki, Finland

## Abstract

Dartagnanis a bounded model checker for concurrent programs under weak memory models. What makes it different from other tools is that the memory model is not hard-coded inside Dartagnanbut taken as part of the input. For SV-COMP’20, we take as input sequential consistency (i.e. the standard interleaving memory model) extended by support for atomic blocks. Our point is to demonstrate that a universal tool can be competitive and perform well in SV-COMP. Being a bounded model checker, Dartagnan’s focus is on disproving safety properties by finding counterexample executions. For programs with bounded loops, Dartagnanperforms an iterative unwinding that results in a complete analysis. The SV-COMP’20 version of Dartagnanworks on Boogiecode. The C programs of the competition are translated internally to Boogieusing SMACK.

## 1 Overview and Software Architecture

DARTAGNAN is a bounded model checker for concurrent programs under weak memory models. It expects as input a program $P$ annotated with a reachability condition $S$, a memory model $\mathcal{M}$, and an unrolling bound $k$. It recursively unwinds all loops in $P$ up to the bound $k$. The unwound program is converted into an SMT formula that symbolically represents all candidate executions. The memory model will filter out some candidates using a second formula, we explain this below. Events of a candidate execution model (instances of) program instructions, like memory accesses, local computations, and conditional/unconditional jumps. Edges model relations between events, including *program order* (the order within a thread), *data-dependencies* (an assigned variable is used within an expression), *reads-from* (matching each read with the write from which it takes its value), and *coherence* (the order in which writes commit to the memory).

A memory model can be understood as a predicate over candidate executions that declares some of them valid. We describe memory models in the CAT language [2]. A memory model is defined as a set of relations (those mentioned

* Jury member.

SC+atomicity

$\mathsf{com} = \mathsf{co} \cup \mathsf{fr} \cup \mathsf{rf}$ $\qquad$ $\mathsf{come} = \mathsf{com} \cap \mathsf{ext}$

$\mathsf{acyclic}\ \mathsf{po} \cup \mathsf{com}$ $\qquad$ $\mathsf{empty}\ \mathsf{rmw} \cap (\mathsf{come}\,;(\mathsf{po} \cup \mathsf{com})^*\,;\mathsf{come})$

**Fig. 1.** CAT model used for SV-COMP'20.

above and others derived as unions, transitive/reflexive closures, compositions, etc.) and constraints over them (emptiness, acyclicity and irreflexivity). Given a memory model, we construct a formula that evaluates to true precisely under the candidate executions that are valid according to the memory model. Figure 1 shows the memory model used for SV-COMP'20. To support atomic blocks, DARTAGNAN adds a specific edge (rmw) for every pair of events between `VERIFIER_atomic_begin()` and its matching `VERIFIER_atomic_end()` or in a `VERIFIER_atomic_` function. We encode atomicity for sequential consistency (SC) as the empty intersection of rwm and paths starting and ending with an external communication (i.e. between different threads). This means once an atomic block starts, external communications with the block are forbidden until all events in the block have been executed.

DARTAGNAN comes with a rich assertion language inspired by HERD [1]. Assertions define inequalities over the values of local and global variables. They can be used freely throughout the code, rather than being limited to the end of the execution. Semantically, our assertions do not stop the execution but record the failure and continue. To achieve this, each instructions `assert(exp)` is transformed to a local computation $\mathtt{f} \leftarrow \mathtt{exp}$ where the fresh variable $\mathtt{f} \in \mathtt{F}$ stores the value of `exp` at the corresponding point of the execution. We refer to the formula $\bigvee_{\mathtt{f} \in \mathtt{F}} \neg \mathtt{f}$ as the reachability condition.

The formula for candidate executions of the program, the formula for validity under the given memory model, and the reachability condition together (in conjunction) yield the SMT encoding of the reachability problem at hand. Any solution to the conjunction corresponds to an execution that is valid according to the memory model and violates at least one assertion. Details on the encoding can be found in [8,9].

DARTAGNAN implements a may-alias analysis to improve pointer precision and a novel relation analysis. The latter technique reduces the SMT encoding to those parts of the relations that might affect the consistency with the memory model, resulting in a considerably smaller formula. Relation analysis improves the performance up to two orders of magnitude [4,5]. We remark that related approaches represent each candidate execution explicitly [1,6]. Thanks to the symbolic representation of executions and static analysis techniques such as relation analysis, DARTAGNAN is often more efficient [4,5].

Figure 2 shows the overall architecture of DARTAGNAN. It reads programs written in the litmus format of HERD [1] or the intermediate verification language BOOGIE [7]. For the competition, C programs are compiled to LLVM and then

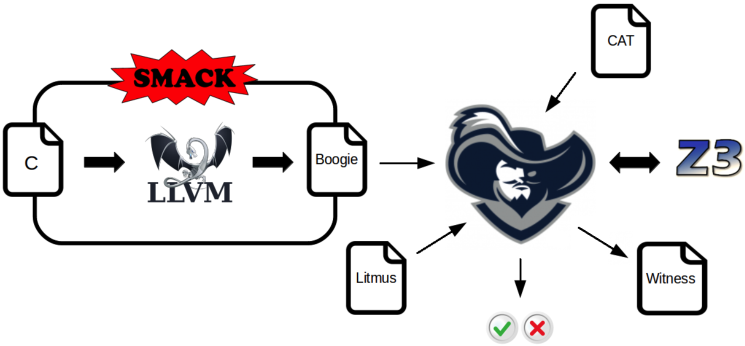

**Fig. 2.** DARTAGNAN's architecture.

translated internally to BOOGIE using the SMACK tool [11]. The SMT solver is Z3 [3]. When a violation is found, DARTAGNAN returns a witness execution.

## 2 Strengths and Weaknesses

The main strength of DARTAGNAN is its fully configurable memory model. Unfortunately, in SV-COMP'20 there is no category for verification tasks under weak memory models. On the SV-COMP'20 benchmarks, DARTAGNAN reports only one incorrect result, being beaten in that aspect only by CPACHECKER, DIVINE, LAZY-CSEQ and YOGAR-CBMC; three of them category winners. The incorrect result is related to the use of pointer arithmetic which is currently not supported by our alias analysis.

Its main strength is also its main weakness: DARTAGNAN's performance cannot quite match that of other verifiers that were developed specifically for sequential consistency. DARTAGNAN performs particularly poor on benchmarks with big atomics blocks. This is the case for most of the verification tasks in the `pthread-wmm` group which represent 83% of the ConcurrencySafety category. The problem is that DARTAGNAN adds rmw edges for all pairs in an atomic block. This results in a large encoding (even using relation analysis) and highly impacts its performance.

## 3 Tool Setup and Configuration

Besides the program to be verified, DARTAGNAN expects a CAT file containing the memory model of interest. For SV-COMP'20, this is the extension of sequential consistency given in Figure 1. The tool is run by executing the following command:

```
$ java -jar dartagnan/target/dartagnan-V-jar-with-dependencies.jar
  -cat <CAT file> -i <program file> [options]
```

Placeholder `V` is the tool version (currently 2.0.5) and `options` is used to configure the unrolling bound, the alias analysis, and the fixpoint encoding. The full list of options can be found on the project website (see Section 4).

To make sure not to miss a violation, the competition version of DARTAGNAN implements an iterative approach. Initially, the bounded model checking algorithm is called with an unrolling bound of one. If it finds a violation or can prove that all loops have been unrolled completely (this is done using unwinding assertions), the verification process terminates with a conclusive answer. If not, DARTAGNAN increases the bound by one and repeats the process. For program with an infinite state space, our tool does not terminate.

DARTAGNAN participates in the ConcurrencySafety category. No specification file is required. The artifact is available on [10]. To reproduce the results of the competition, the tool can be executed with the following wrapper script:

```
$ Dartagnan-SVCOMP.sh <program file>
```

## 4 Software Project and Contributors

The project home page is https://github.com/hernanponcedeleon/Dat3M. DARTAGNAN is open source software distributed under the MIT license.

*Acknowledgement:* We thank Dirk Beyer and Philipp Wendler for their help during the process of integrating DARTAGNAN into the competition framework. We also thank Natalia Gavrilenko for her contributions to the development of the bounded model checking engine of the tool [4,5].
